# Detection of specific HPV subtypes responsible for the pathogenesis of condylomata acuminata

**DOI:** 10.1186/1743-422X-10-137

**Published:** 2013-05-01

**Authors:** Matthew G Hawkins, David M Winder, Siolian L R Ball, Katie Vaughan, Christopher Sonnex, Margaret A Stanley, Jane C Sterling, Peter K C Goon

**Affiliations:** 1 Department of Pathology, University of Cambridge, Tennis Court Road, Cambridge, CB2 1QP, UK; 2 Department of Genito-Urinary Medicine, Box 38, Addenbrooke’s Hospital, Hills Road, Cambridge, CB2 0QQ, UK; 3 Department of Medicine, University of Cambridge, Box 157, Addenbrooke’s Hospital, Hills Road, Cambridge, CB2 0QQ, UK

**Keywords:** HPV types, Warts, Laser-capture and microdissection, Quantitative PCR, DNA, mRNA

## Abstract

**Background:**

The low-risk human papillomavirus types 6 and 11 are responsible for approximately 90% of anogenital wart cases, with approximately 190,000 new and recurrent cases reported in the UK in 2010. The UK has recently selected the quadrivalent HPV vaccine, which conveys protection against both HPV6 and HPV 11, as part of its immunisation programme for 2012 and it is expected that this will reduce disease burden in the UK. The aims of the study were to evaluate current strategies used for the monitoring of HPV infection in genital warts and to assess the suitability of laser-capture microdissection (LCM) as a technique to improve the understanding of the natural history of HPV types associated with genital wart lesions.

**Methods:**

DNA and RNA were extracted from whole wart, surface swabs and LCM sections from 23 patients. HPV types present were determined using the Linear Array HPV Genotyping Test (Roche), with HPV DNA viral load and mRNA expression investigated using qPCR and qRT-PCR, respectively.

**Results:**

Results indicated that swabbing the surface of warts does not accurately reflect potential causative HPV types present within a wart lesion, multiple HPV types being present on the surface of the wart that are absent in the lower layers of tissue isolated by LCM. Although it was shown that HPV DNA viral load does not directly correlate with HPV mRNA load, the presence of both DNA and mRNA from a single HPV type suggested a causative role in lesion development in 8/12 (66.6%) of patients analysed, with dual infections seen in 4/12 (33.3%) cases. HPV 6 and HPV 11 were present in more than 90% of the lesions examined.

**Conclusions:**

Surface swabbing of warts does not necessarily reflect the causative HPV types. HPV type specific DNA and mRNA loads do not correlate. HPV 6 and 11 were likely to be causally involved in over 90% of the lesions. Dual infections were also found, and further studies are required to determine the biological and clinical nature of dual/multiple infections and to establish the relationship of multiple HPV types within a single lesion.

## Background

Human papillomavirus (HPV) is the aetiological cause of anogenital warts. Anogenital wart disease is the most common sexually transmitted disease, affecting millions worldwide every year. In 2010, approximately 190,000 new and recurrent cases were reported through GU clinics in the UK [1], with the low-risk HPV types (LR-HPV) 6 and 11 predominating in the pathogenesis of the common anogenital wart, or condyloma acuminatum [2]. The licensing of the prophylactic quadrivalent vaccine Gardasil (Merck) in 2007, which conveys protection against HPV types 6 and 11 in addition to the high-risk types HPV 16 and 18, has now raised the real prospect of reducing the disease burden of anogenital warts in society. It has already been shown that a high coverage of eligible females (approximately 80%) has resulted in a 73% fall in incident genital warts in Australian women since 2007 [3]. These observations indicate a potentially large impact on disease burden elsewhere. The UK has recently selected the quadrivalent HPV vaccine as part of its immunisation programme for 2012. The annual cost of wart disease diagnoses and treatments to the NHS in the UK is thought to be as high as £52.4 million [1].

Most epidemiological studies on wart disease have involved swabbing of the surface of lesions with subsequent HPV typing being undertaken on the extracted DNA [4-7]. Interestingly, studies have also documented that multiple infections with several different HPV subtypes are common [8-10]. In the cervix it has recently been shown that a lesion is almost always associated with a single HPV type, although there can be several lesions abutting each other, and thus give the impression of a single lesion [11]. Wart lesions may also reflect such a pattern, but a thorough analysis in a similar manner has not been previously reported. The question therefore remains, whether the multiplicity of HPV types reported from genital wart swabs is genuinely reflective of the types causally associated with the lesion.

The aim of this study was to determine whether surface swabbing of warts accurately reflects the HPV type or types causing the epidermal proliferation. Surface contamination with other HPV types was determined by comparing the HPV types found in swab samples to those present in whole wart tissue, using the Linear Array HPV genotyping test (Roche). Laser-capture microdissection (LCM) was then used to isolate the surface and the basal areas for further analyses, permitting the identification of potential candidate HPV types responsible for the wart lesion. Furthermore, HPV DNA and mRNA, thought to be the “gold-standard” by which HPV type is linked with cervical lesion progression [12-14], were quantified and compared with the Linear Array data. RNA analysis was used as the final arbiter of HPV activity within the wart, based on the hypothesis that a transcriptionally active HPV type is associated with actual lesion causation. Messenger RNA analysis for HPV transcriptional activity also eliminates the problem of surface contamination with “bystander” virus, as HPV gene transcription is fully dependent on cellular replication machinery [15]. The suitability of surface swabbing as a method for the detection of HPV types present in anogenital warts is discussed.

## Results

**Figure 1:**
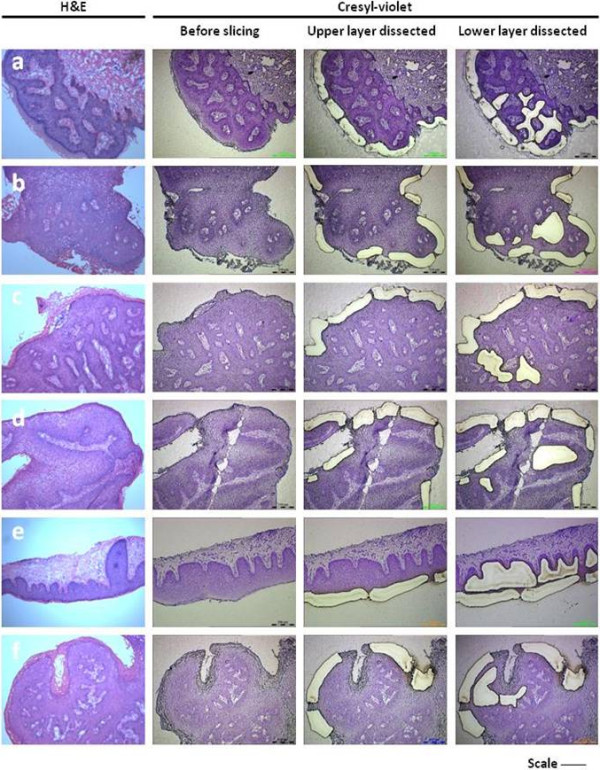
**Capture of wart tissue using Laser-Capture Microdissection. **The series of photographs identifies the sequential extraction of lower and upper layers within wart tissue. Tissues a – f are histological specimens of wart tissue stained with either H&E (left-most column) or cresyl violet (other columns). Scale bar for image = 300 um, except tissue e = 250 um.

HPV typing was performed by utilising DNA extracted from swabs and whole wart tissue in 23 samples, and the upper and lower layers of wart tissue dissected out using laser-capture microdissection (LCM) in 16 out of 23 patients. Figure 1 shows typical examples of the use of laser-capture microdissection (LCM) for obtaining tissue specific to the areas intended for analysis. LCM was used to determine the likelihood of surface contamination by dissecting out the upper and lower layers of wart tissue. These layers acted as comparative controls for surface contamination, both positive and negative, respectively – as the upper layer includes the potentially contaminated external wart surface (including *stratum corneum* and *stratum granulosum*) and the lower layer includes the uncontaminated *stratum spinosum* and *stratum basale*.

The prevalence of HPV types present in the whole wart, swabs, and the upper and lower microdissected layers, classified into either low-risk or high-risk (Table 1). The five most common HPV types detected via the Linear array method in wart tissue were HPV 6 (82.6% of all samples), HPV 16 (43.5%), HPV 45 (34.8%), HPV 11 (17.4%) and HPV 42 (13.0%). In swabs, the five most common types of HPV present were HPV 6 (78.2%), HPV 16 (47.8%), HPV 45 (26.1%), HPV 51 (21.7%) and HPV 84 (17.4%). In the lower layers of warts, HPV 6 was by far the dominant type and was found in 75.0% of samples analysed. HPV 45 was next most common (31.3%) and HPV 52 was third (18.8%). In the upper layers of tissue, HPV 6 was again dominant (75.0%), followed by HPV 16 (37.5%), and third was HPV 81 (31.3%).

**Table 1:** HPV type identification in whole wart tissue, swabs, lower and upper layers of wart tissue

**Sample #**	**Whole wart tissue**		**Swab**		**Lower layer**		**Upper layer**	
	**Low risk**	**High risk**	**Low risk**	**High risk**	**Low risk**	**High risk**	**Low risk**	**High risk**
1	6, 42	59	6, 42, 84, CP*	58, 59	11	-	11, 81	16, 45
2	6	16	6, 55	16	6	45, 52	6	-
3	6	16	6	16	6	18, 45, 52	6	16, 52
4	6	-	6, 84	53, 59	6, 81	45	6, 81	16
5	6	16, 45, 53	6	16, 45, 52, 53, 66	6	53	6	53
6	6	-	6	73	6	-	6	-
7	6, 11, 81	-	6	16, 18	6	-	6	-
8	6	33, 52	6	16	6	-	6, 81	-
9	6	16	6	16	6	-	6	-
10	6	-	6	45, 52	6	-	6	16, 18, 31, 52
11	6, 55	16, 45	55	51	55	-	55	53
12	11, 81	16	11, 81	16	11, 81	16	11, 81	16
13	6	16	6	16	6	16, 45	6	-
14	6	45	6	45, 51	6	45, 52	6	-
15	6, 55	16, 45, 52	6	-	6	-	6	16
16	55	16, 31, 45	CP*	16, 18, 31, 45, 51, 66	54	31	54, 81	31
17	11	-	11	-	**		**	
18	6	18, 33, 45, 52	6	16	**		**	
19	6	45	6, 84	45	**		**	
20	6, 42	-	6	18	**		**	
21	6, 42	45, 51	6, 42	45, 51, 67, 82	**		**	
22	11	16	11, 84	-	**		**	
23	6	51	6, 62	16, 51	**		**	

Roche linear array genotyping was performed on 23 individual patients using 50 ng DNA, and the HPV types identified. CP* abbreviated for CP6108 and is now known as HPV 89. **Insufficient DNA.

There was very good concordance between HPV types detected between swabs and whole tissue, particularly for HPV 6, HPV 11 and HPV 31 (κ = 0.862, 0.832 and 1.000, respectively). Furthermore, substantial agreement (0.80 ≥ κ > 0.60) (κ = 0.646) occurred for both HPV types 59 and 81. No concordance was observed (κ ≤ 0) for HPV 16, HPV 52, HPV 53 or HPV 54. Remaining HPV types exhibited at least fair agreement (κ > 0.20). Overall concordance for all types found between whole wart tissue and swabs was κ = 0.574 (moderate agreement).

The overall concordance for all HPV types detected between upper and lower layers of wart tissue was κ = 0.632 (substantial agreement). However, when compared with whole wart tissue, both upper and lower layers only exhibited moderate agreement (κ = 0.490 and 0.538, respectively). Furthermore, concordance estimates for HPV 6 was reduced from κ = 1.000 (perfect agreement in upper vs lower analyses) to substantial (κ = 0.600 for both wart vs upper and wart vs lower). HPV 11 was also reduced from κ = 1.000 to moderate (κ = 0.429 for both). This suggests that the LCM tissue is not representative of its corresponding whole wart tissue in terms of HPV types detected.

HPV types were subsequently classified as surface contamination, undetermined or infection candidates, dependent on the presence or absence of HPV in the tissue samples analysed (Table 2). These categories were derived from the assumption that HPV types found in the interior or basal areas of warts are likely to be involved in the pathogenesis of the wart lesion. Hence, infection candidates are those subtypes identified from [lower layers + (whole wart – upper layers)]. Likely surface contaminants are [(upper layers – lower layers) + (swab + whole wart – lower layers) + (swab – whole wart)].

**Table 2:** Identification of potential surface contaminants and infection candidates

**Classification of HPV type as containing, infection candidate or undetermined**			
**Sample #**	**Surface contamination**	Undetermined*	**Infection candidates**
1	16, 45, 58, 81, 84, CP^$^	6, 42, 59	11, 81
2	55	16	6, 45, 52
3	16	-	6, 18, 45, 52
4	16, 53, 59, 84	-	6, 45, 81
5	52, 66	16, 45	6, 53
6	73	-	6
7	16, 18, 81	11	6, 42, 59
8	16, 81	33, 52	6
9	-	16	6
10	16, 18, 31, 45, 52	-	6
11	51, 53	6, 16, 45	55
12	-	-	11, 16, 81
13	-	-	6, 16, 45
14	51	-	6, 45, 52
15	16	45, 52, 55	6
16	18, 51, 66, 81, CP^$^	16, 45, 55	31, 54
17**	-	11	-
18**	16	6, 18, 33, 45, 52	-
19**	84	6, 45	-
20**	18	6, 42	-
21**	67, 82	6, 42, 45, 51	-
22**	84	11, 16	-
23**	16, 62	6, 51	-

Data from each sampling technique was compared, and likely contaminants were identified and potential infection candidates were determined: - infection candidates are those subtypes identified from [lower layers + (whole wart – upper layers)]. Likely surface contaminants are [(upper layers – lower layers) + (swab + whole wart – lower layers) + (swab – whole wart)]. *Absence of HPV type in swab, lower and upper layers cannot rule out possibility of contamination (since wart tissue contains both contaminants and non-contaminants). ** Absence of LCM cannot confirm any HPV type as truly non-contaminant. CP^$ ^abbreviated for CP6108 and is now known as HPV 89.

Quantitative DNA and mRNA analyses for HPV E6 were performed for HPV 6, HPV 11, HPV 16, HPV 45 and 52 (Table 3). These HPV types were the most prevalent types in the list of infection candidates, with the exception of HPV 11. HPV 11 was chosen because it is known to be a common type associated with warts. HPV 6 and HPV 11 were seen to be the dominant types, in terms of both DNA viral loads and mRNA expression. Four lesions exhibited mixed RNA expression, implying multiple actively replicating HPV types in the lesion: sample 4 (HPVs 6 and 11), sample 9 (HPVs 6 and 11), sample 11 (HPVs 6 and 11) and sample 12 (HPVs 11 and 16). No correlation between DNA viral load and mRNA expression was seen in wart tissue for HPV 6, which was the predominant type found in this cohort (p = 0.3034, Spearman’s Rank Correlation Coefficient, 2 tailed test).

**Table 3:** Comparing HPV DNA viral loads and RNA levels in whole wart tissue

**Sample**	**HPV DNA viral load (copies/cell)**					**HPV RNA level (copies/μg RNA**				
	**HPV6**	**HPV11**	**HPV16**	**HPV45**	**HPV52**	**HPV6**	**HPV11**	**HPV16**	**HPV45**	**HPV52**
1	45.71	0	0*	0*	0	191.25	0	0*	0*	0
2	213.05	0.01	0.04	0	0	370	0	0	0	0
3	101.47	0	0*	0	0	0	0.34	0*	0	0
4	333.88	0	0*	0.03	0.05	186.77	26.88	0*	0	0
5	10974.83	0	115.18	0.35	0*	45.53	0	0	0	0.04*
6	13303.58	0	0.05	0	0	2047.98	0	0	0	0
7	218.29	0	0.03*	0	0	900.90	0	0*	0	0
8	604.56	0.01	0.03*	0	0	1341.49	0	0*	0	0
9	161.60	0	0.03	0	0	70.61	20.81	0	0	0
10	171.66	0	0.01*	0*	0*	5883.86	0	0*	0*	0*
11	279.68	0.01	0.18	0	0.02	856.98	43.52	0	0	0
12	0.01	4.31	172.88	0	0	0	51.15	17.20	0	0
13	571.85	0	0	0	0	-	-	-	-	-
14	48.47	0	0	0	0	-	-	-	-	-
15	0.22	0	0.41*	0	0	-	-	-	-	-
16	0	0	0.10	0	0.01	-	-	-	-	-
17	0	3.07	0.02	0	0	0	439.28	0	0	0
18	171.74	0	0.04*	0	0	0	0	0*	0	0
19	422.87	-	-	-	-	-	-	-	-	-
20	36.35	0.01	0	0	0	-	-	-	-	-
21	206.25	0.09	0.04	0	0	-	-	-	-	-
23	22.61	0.06	0*	0	0	-	-	-	-	-

HPV DNA viral loads (viral copies/cell) and RNA transcript levels (copies/μg RNA) were obtained by qPCR and qRT-PCR, respectively. (-) indicates that there was insufficient material for analyses. Samples containing HPV types previously determined as a likely contaminant (refer to Table 2) have been highlighted with an asterisk (*) and excluded from any further analysis.

Potential infection candidates, identified by subtracting probable and confirmed contaminant types, were then compared to the HPV types with confirmed expression from the mRNA analysis (Table 4). Poor correlation was found between potential infection candidates and HPV types with mRNA expression. Only 10/17 (58.8%) candidates from the RNA positive list were found to be identified in the infection candidate list. In sample 1, HPV 6 was found in the undetermined category. In four cases (samples 3, 4, 9 and 11), HPV 11 was not found by the Linear Array system. These cases illustrate the fact that use of mRNA expression (as opposed to pure DNA analyses alone) will aid final conclusions. Finally, only 76.5% of candidates identified by elevated mRNA levels were included in the list of potential or undetermined infection candidates.

**Table 4:** Identification of candidate HPV types causing wart lesion development

**Sample #**	**Infection candidates (refer Table **2**)**	**Potential cause of lesion (RNA analyses)**
1	11, 81	6^b^
2	6^a^, 45, 52	6^a^
3	6, 18, 45, 52	11^d^
4	6^a^, 45, 81	6^a^, 11^d^
5	6^a^, 53	6^a^, 52^c^
6	6^a^	6^a^
7	6^a^, 42, 59	6^a^
8	6^a^	6^a^
9	6^a^	6^a^, 11^d^
10	6^a^	6^a^
11	55	6^b^, 11^d^
12	11^a^, 16^a^, 81	11^a^, 16^a^
17**	-	11^b^

Identification of candidate HPV types causing warts by HPV E6 RNA as arbiter of type involvement compared to laser capture & microdissection (LCM) with DNA typing.*Absence of HPV type in swab, lower and upper layers cannot rule out possibility of contamination (since wart tissue contains both contaminants and non-contaminants). **Absence of LCM cannot confirm any HPV type as truly non-contaminant. CP^$ ^abbreviated for CP6108 and is now known as HPV 89. HPV types **correctly identified as infection candidates** are highlighted by superscript (**a**); HPV types identified as potentially causing lesion development in the **‘undetermined’ **category are highlighted by superscript (**b**); HPV types **incorrectly identified as surface contaminants** have been highlighted by superscript (**c**); HPV types potentially causing wart lesion development but **not detected using Linear Array** are highlighted by superscript (**d**).

## Discussion

Anogenital warts is the most common sexually transmitted disease in the world, and is also a marker of transmission of high-risk HPV types, such as HPV 16 and HPV 18, due to similar infection routes. Diagnosis with genital warts is associated with a long-term increased risk of anogenital and head and neck cancers [16]. The monitoring of clinically obvious disease, such as anogenital warts, therefore has an important bearing upon overall HPV disease in the community at large. It is accepted that HPV 6 and HPV 11 are the dominant types in the pathogenesis of anogenital warts and in the upper respiratory tract equivalent, respiratory papillomatosis [17]. However, it is also known that other benign or low-risk types have been implicated in the causation of anogenital warts [5,10,18-20]. One potential confounding factor in these studies is that surface swabbing of lesions for HPV DNA, a commonly used method to define the HPV types involved, is susceptible to contamination with unrelated HPV types.

In this study, laser-capture microdissection was used to facilitate the delineation of HPV types likely to be surface contaminants from those likely to be involved in the aetiology of the wart lesion. Subsequent real-time PCR analyses for both E6 DNA and E6 mRNA allowed their suitability for monitoring purposes to be determined. The use of simple equations, based on the premise that HPV types identified from within the deeper epithelium of the wart are more likely to be causal, allowed “infection candidates” to be identified. Messenger RNA detection for five common HPV types found in this cohort was then used to identify the most likely candidate to be involved in actual lesion pathogenesis. HPV 6 was a more frequent cause of lesions than HPV 11. Interestingly, the majority of HPV types identified as surface contaminants were high-risk types (75%), with the remainder being identified as low-risk types (25%). The majority of contaminant HPV types featured only once or twice in the samples (incidence <8%). HPV 16 was the most common surface contaminant (25% of wart lesions). Interestingly, many of the surface contaminants detected via the LCM and typing analyses were high-risk types and we speculate that perhaps high-risk types have an innate survival advantage over many low-risk types (with the exception of HPV 6 and 11, which are very successful wart developers) and this aids their transmission rates.

The incidence of multiple infections detected in wart studies ranges from 33.8% to 40.1% when using only swabbing techniques [10,21], as well as 21.7% when using fresh wart tissue specimens [22]. These infections were a mixture of high-risk and low-risk types (range: 2–5 types) but usually contained HPV 6 or 11, or both. In our cohort, 28.6% anogenital warts were co-infected with active (E6 mRNA+) HPV types, similar to the published estimates discussed above. However, it is important to note that this statistic is two-fold less than the co-infection levels observed for the DNA detection methods and without exclusion of surface contamination. Therefore existing data concerning multiple infections may be an overestimate. On the other hand, it is possible that the suspected multiple infections are in fact two different lesions growing together and appearing as a single lesion, as has been shown for the cervix [11]. Quint et al. demonstrated that for the cervix, a single lesion is most often associated with a single HPV type. Where multiple types were found, careful laser-capture microdissection of histopathological sections found that different types were associated with several abutting lesions, but with the over-riding conclusion that each type caused a single lesion. There are no data to suggest that two or more types of HPV can infect a single cell and this study does not address that question.

Moderate concordance exists between whole wart tissue and corresponding swabs in terms of HPV detection (κ = 0.574), with high concordance levels for HPV types 6, 11, 31, 59 and 81. This illustrates that anogenital swabs can be used with reasonable reliability to determine HPV types detected in whole wart tissue. However, this study has also shown that HPV DNA viral load estimates from swab samples do not reflect that which is observed in paired wart tissue. Furthermore, the elucidation of surface contamination highlights potential limitations with non-adjusted HPV prevalence data obtained from swab samples [5,10,18,23] and also disagrees with the claim that HPV identification in swabs can be used for analysing clinical changes in a specific lesion. It would thus seem apparent that careful consideration may be needed concerning the existing literature on HPV prevalence in anogenital warts. However, the use of swabs for monitoring the effects of vaccination and treatment [20] is likely to remain valid.

Interestingly, the lack of correlation between DNA viral loads and mRNA expression suggests that measuring viral DNA levels is not representative of viral activity within the wart. It is possible that the efficiency of the viral transcription machinery differs between individual viruses. Alternatively, it is possible that either the detection sensitivities for the viral DNA/mRNA components using the methods employed vary or there is a differing level of viral E6 gene expression during various stages of the virus replication lifecycle.

The importance of HPV 6 and HPV 11 in the pathogenesis of classic condylomata acuminata has been demonstrated here, and has been deemed to be causal in over 90% of this small cohort of 23 patients. These data therefore support published studies in the literature. There has however, been little RNA work performed in wart disease which could serve to either support or refute this finding, only previous publications which quote the 90% level of causation for these two dominant types [24,25]. It may be that the total amount of clinical wart disease prevented via the L1 prophylactic vaccine will be similarly high or even higher. Furthermore, if herd immunity of human populations around the world can be achieved utilising currently available L1 prophylactic vaccines, rare diseases such as recurrent respiratory papillomatosis (RRP) will also be reduced but this will require long-term population surveillance.

The detailed and thorough molecular work involved in this small study has revealed that, although multiple infections are apparently a frequent occurrence when determined by swabbing wart lesions, the actual numbers of types potentially involved in lesion pathogenesis is low. This is an important and potentially confounding factor in all studies that involve only the surface swabbing of wart lesions. We also show for the first time that laser capture microdissection can be utilised successfully to assist in objective analyses of the aetiology of anogenital warts, but there was poor correlation with detected RNA levels. This technique is expensive and requires specialised skills and is likely to remain a research tool only.

In this study, samples 4, 9, 11 and 12 demonstrated evidence of dual infection. Interestingly, HPV 16 was shown be elevated for both DNA and mRNA in sample 12, which is evidence of active replication in that lesion. In four cases (samples 3, 4, 9 and 11), HPV 11 was not found by the Linear Array system. The reason for this is not known but may be related to sensitivity levels. The manufacturer has found that their product is able to detect HPV 11 plasmids at a level of 900 copies/ml and it is possible that the virus is present with a DNA quantity lower than this detection threshold. The data shows that HPV 6 or HPV 11 appear to be involved causally in a majority of lesions clinically diagnosed as typical condylomata acuminata. The data also suggests a potential role for HPV 16 or other types in isolated cases of wart disease and this hypothesis needs to be explored in further studies.

## Methods

### Study samples

Clinical samples were obtained from patients attending the Departments of Gynaecological Oncology and Dermatology, Addenbrooke's Hospital, Cambridge, UK. Ethical approval was obtained from the local research ethics committee (Cambridgeshire 3 REC reference no: 07/H0306/127) and all patients gave written informed consent for the use of these samples for this study. Anogenital warts were excised as part of treatment and dissected into five equal segments using sterilised scalpel blades, following which they were frozen in liquid nitrogen until DNA extraction. Prior to excision, a concomitant Dacron swab sampled the anogenital wart surface. The swab was agitated in PBS and discarded. The cells obtained from the swab sample were suspended in PBS and stored at -20°C prior to DNA extraction.

### Laser-capture microdissection (LCM)

One segment of sample was removed from liquid nitrogen and thawed on ice. Fresh unfixed tissue segments were embedded in optimal cutting temperature (OCT) compound. Multiple 7 μm OCT-embedded sections were sliced and mounted to PEN (polyethylene naphthalate) membrane coated slides (Leica Microsystems Ltd., UK), being stored at -70°C prior to LCM. An additional section of tissue was sliced onto Biobond-coated Superfrost slides (VWR International, UK) and stained with haematoxylin and eosin (H&E) in order to allow tissue strata to be identified.

Prior to LCM, slide-bound tissue sections were stained with cresyl violet. Tissue dehydration was performed by a series of alcohol baths for 1 minute (70, 95 and 100% ethanol). Lipids were removed from the tissue by bathing in 1:1 chloroform:ethanol for 3 minutes. The tissue was then rehydrated (1 minute in 95% then 70% ethanol and H_2_O) and stained with 0.4% cresyl violet solution for 10 seconds and immediately washed with distilled water to remove any residual staining medium. Sections were allowed to dry prior to transportation on ice.

LCM of frozen tissue sections was performed using Leica LMD7000 Laser Microdissection System (Leica Microsystems Ltd., UK). The slide was inverted and fixed under the microscope where a 0.5 μm laser beam was used to dissect selected areas of tissue. Dissected tissue was allowed to fall into a collecting tube, containing 40 μl PBS, placed underneath the inverted slide as summarised in video format, http://www.jove.com/index/Details.stp?ID=309[26]. The collection tube was stored on ice until DNA extraction. Dissections were performed in triplicate.

### DNA extraction and HPV typing

Samples of whole wart tissue were inserted into a FastPrep lysing matrix tube (MP Biomedicals Europe, Illkirch, France) and disrupted in a Bullet Blender™ (Next Advance Inc., Averill Park, USA) prior to genomic DNA (gDNA) extraction [27]. gDNA was extracted from PBS containing debris from swabs using the DNeasy Blood and Tissue Kit (QIAGEN Ltd., Crawley, UK) according to a modified protocol [28]. gDNA was purified from LCM samples using the DNeasy Blood and Tissue Kit (QIAGEN Ltd., UK), according to the manufacturer's instructions.

The Roche Linear Array HPV Genotyping Test (LA HPV GT - Roche Diagnostics Ltd., Burgess Hill, UK), allowing detection of 37 high- and low-risk HPV types, was carried out according to the manufacturer’s instructions [29].

### mRNA extraction and cDNA synthesis from whole wart tissue

RNA was extracted from whole wart tissue, as previously described [8]. Briefly, tissue was suspended in 200 μl Trizol™ (Invitrogen, Paisley, UK) in a lysing matrix D tube (MP Biomedicals, Solon, OH) and pulverized using a Bulletblender™ machine (Next Advance, Averill Park, NY). RNA was precipitated with isopropranol and resuspended in 50 μl H_2_O. After DNAse I digestion, RNA was recovered by column purification using PureLink™ RNA extraction kit (Invitrogen) following manufacturer’s instructions and a maximum of 5 μg reverse transcribed using Bioscript™ (Bioline, London, UK) after preincubation with random hexamer primers for 5 min at 65°C. Reactions were performed as follows: 25°C for 10 min, 42°C for 60 min and 70°C for 15 min. No-RT controls were performed without the addition of enzyme.

### qPCR for HPV E6

The primers and probes targeting the HPV E6 gene for types 6, 11, 16, 18, 45 and 52, together with human GAPDH (glyceraldehyde 3-phosphate dehydrogenase) and β-globin are described elsewhere (20–23). All qPCR reactions were performed in 20 μl containing 1x PCR buffer (Qiagen, UK), 4.5 mM MgCl_2_ (Qiagen, UK), 375 μM dNTPs (Roche, UK), 100 nM primer pairs and probe (Sigma Genosys, UK), 0.5 units Hotstart Platinum Taq (Qiagen, UK) and template.

Relative cellular mRNA for HPV E6 was determined by normalizing HPV values, obtained following qPCR of wart mRNA, with TBP (TATA-Box binding protein), YWHAZ (tyrosine 3-mono-oxygenase/tryptophan 5-mono-oxygenase activation protein, zeta polypeptide) and HMBS (hydroxymethylbilane synthase) using primers and probes that have been previously described (24–26).

qPCR amplification was performed using Rotor-Gene 3000 (Corbett Life Sciences). Cycling conditions were as follows: an initial activation/denaturation step at 95°C for 15 min, then 40 cycles of 95°C for 15 sec and 60°C for 60 sec, with acquisition of fluorescent signal at 60 sec. Data was analysed using the Rotor-Gene analysis software (version 6.1). The quantification of HPV DNA viral load and mRNA expression in samples was accomplished through the use of a standard curve incorporated into each experiment.

*The choice of E6 DNA and mRNA as a target for our qPCRs for DNA and RNA viral loads was taken because E6 and E7 are crucial early proteins active in all alpha-group papillomavirus replication, DNA synthesis, abortive infections and productive infections but not in latent infections [30-32].

### Statistical methods

The concordance and agreement between samples for each HPV type detected was calculated. Kappa values of 0–0.2 (slight), 0.21-0.4 (fair), 0.41-0.6 (moderate), 0.61-0.8 (substantial) and 0.81-1.0 (almost perfect) indicated the level of agreement between the methods used [33]. Statistical analyses were performed with SPSS software.

## Abbreviations

LCM: Laser-capture microdissection; qPCR: Quantitative PCR; qRT-PCR: Quantitative reverse-transcribed PCR.

## Competing interests

MAS is consultant to Sanofi Pasteur-MSD, Lyon, France and PKCG & MAS were in receipt of an unrestricted educational grant. PKCG was previously consultant to SP-MSD two years ago. MAS is also consultant to Merck Research Laboratories, Westpoint, USA, and GSK Biologicals, Rixensart, Belgium. CS has been in receipt of travel funds from SP-MSD and GSK.

## Authors’ contributions

MH carried out the molecular genetic studies, LCM, performed the statistical analysis and helped draft the manuscript. DW, SB, KV helped perform the molecular genetic studies. CS, MAS participated in the drafting of manuscript. JCS participated in the design of the study and helped draft the manuscript. PG conceived of the study, and participated in its design and coordination and wrote the manuscript. All authors read and approved the final manuscript.
